# Dietary therapies interlinking with gut microbes toward human health: Past, present, and future

**DOI:** 10.1002/imt2.230

**Published:** 2024-08-10

**Authors:** Jiali Chen, Jiaqiang Luo, Sjaak Pouwels, Beijinni Li, Bian Wu, Tamer N. Abdelbaki, Jayashree Arcot, Wah Yang

**Affiliations:** ^1^ Department of Food Science and Engineering, College of Life Science and Technology Jinan University Guangzhou China; ^2^ School of Chemical Engineering, Faculty of Engineering, UNSW Sydney Kensington New South Wales Australia; ^3^ Department of Surgery Marien Hospital Herne, University Hospital of Ruhr University Bochum Herne North Rhine‐Westphalia Germany; ^4^ College of Future Technology The Hong Kong University of Science and Technology Guangzhou China; ^5^ Department of General Surgery II The First People's Hospital of Yunnan Province, The Affiliated Hospital of Kunming University of Science and Technology Kunming China; ^6^ Department of General Surgery Alexandria University Faculty of Medicine Alexandria Governorate Egypt; ^7^ Department of Metabolic and Bariatric Surgery The First Affiliated Hospital of Jinan University Guangzhou China

## Abstract

Overview of personalized dietary therapies. This flow chart exhibits the future prospect for integrating human microbiome and bio‐medical research to revolutionize the precise personalized dietary therapies. With the development of artificial intelligence (AI), incorporating database may achieve personalized dietary therapies with high precision.

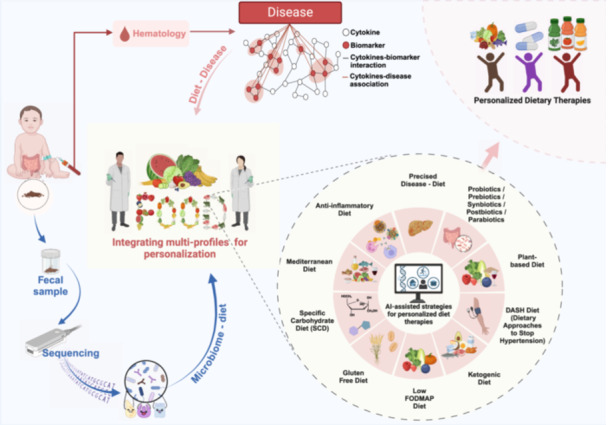

## GLOBAL CHALLENGES ON DIET PATTERN CHANGE

With accelerated industrialization and urbanization, traditional diets are gradually replaced by high intakes of meat, refined sugars, and fats. This dietary shift poses significant risks and challenges to human health. This “modern dietary pattern” has led to the global prevalence of chronic noncommunicable diseases (NCDs) including obesity, diabetes, and cardiovascular disease, resulting in a reduction in life expectancy [1]. High consumption of ultra‐processed food may influence food overall intake with high energy density through disrupting gut‐brain signaling [2]. With the optimization of living conditions and increasing public concern for dietary safety and health, improving health through different dietary therapies according to individual physiological needs deserves further exploration and in‐depth research.

## CORRELATION OF DIETARY THERAPIES AND GUT MICROBES: IMPACT ON HUMAN HEALTH

**Figure 1:**
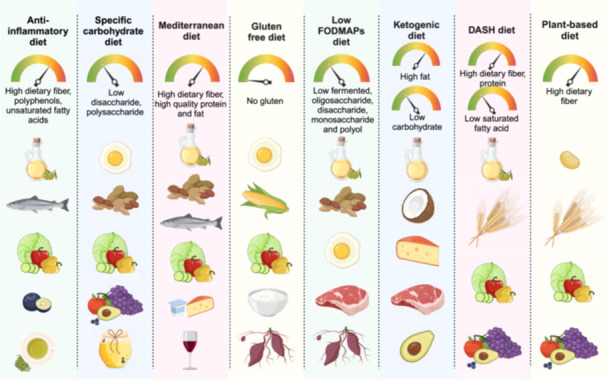
Characteristics illustration of popular dietary patterns. Anti‐inflammatory diet, specific carbohydrate diet, Mediterranean diet, gluten‐free diet, low‐fermented, oligosaccharide, disaccharide, monosaccharide and polyol (FODMAPs) diet, ketogenic diet, dietary approaches to stop hypertension (DASH) diet, and plant‐based diet are the main dietary pattern with various health‐aware food recommendation. The representative types of food are listed for each dietary pattern. Anti‐inflammatory diet, Mediterranean diet, DASH diet, and plant‐based diet are similarly characterized with high dietary fiber. The specific carbohydrate diet is rich in food with low disaccharide and polysaccharide. Gluten‐free diet is defined as being strict exclusion of gluten for life. Low FODMAPs diet is defined as food with low fermented, oligosaccharide, monosaccharide, and polyol. The ketogenic diet is characterized as low carbohydrates and high fats. Figure created with BioRender.com.

Gut microbiota is the largest and most diverse microbial community among the resident microorganisms in the human body, and its specific composition and diversity have been associated with many health conditions. They can play an important role in the host's health. It is well known that “You are what you eat”, meaning that long‐term dietary habits can impact the composition and activity of the body's gut microbes from a scientific point of view. The modern dietary pattern, characterized by a Western‐style high‐fat diet (HFD), has been shown to negatively impact the gut microbiota. This is evidenced by increased populations of *Erysipelotrichaceae*, facultative anaerobic bacteria, and opportunistic pathogens [3]. Host–microbe interactions produce metabolites such as bile acids, lipopolysaccharide (LPS), short‐chain fatty acids (SCFAs), and trimethylamine N‐oxide (TMAO), playing a critical role in host health. HFD alters the metabolism of gut microbiota and accelerates the development of NCDs through Farnesoid X receptor (FXR), Takeda‐G‐protein‐receptor‐5 (TGR5), nuclear factor‐kappa B (NF‐*κ*B), peroxisome proliferator‐activated receptor *γ* (PPAR‐*γ*), and protein kinase RNA–like endoplasmic reticulum kinase (PERK) signaling [3]. Multiple dietary patterns have been proposed to regulate human health with diverse mechanisms. As shown in Figure 1, anti‐inflammatory diet, specific carbohydrate diet, Mediterranean diet, gluten‐free diet, low‐fermented, oligosaccharide, disaccharide, monosaccharide and polyol (FODMAPs) diet, ketogenic diet, dietary approaches to stop hypertension (DASH) diet, and plant‐based diet were found as the main dietary patterns. An anti‐inflammatory diet of unrefined and minimally processed foods has been shown to significantly reduce inflammation compared to the modern dietary pattern of consuming ultra‐processed foods. An anti‐inflammatory diet rich in dietary fiber, polyphenols, and unsaturated fatty acids can modulate the composition of gut microbes and increase the production of SCFAs with beneficial effects on health [4]. A high‐fat but very low‐carbohydrate ketogenic diet has been reported that it could effectively inhibit the growth of Bifidobacteria, interfering the production of Th17 cells [5]. The high‐protein and low‐carbohydrate Paleolithic diet, commonly used for weight loss in Western societies, has been used to treat inflammatory bowel disease clinically, although the impact of Paleolithic diet on the microbiota remains unclear [6]. Specific dietary patterns such as low FODMAPs, specific carbohydrate diet (SCD), and gluten‐free diets (GFD) have been considered to be effective therapeutic options in some pathological conditions such as irritable bowel syndrome (IBS), and celiac disease (CD). Low‐FODMAPs diets cause significant changes in the composition of the gut microbiota, such as leading to lower levels of Bifidobacteria, however, more studies are needed to further understand whether these changes are harmful to health [7]. GFD also induces changes in the gut microbiota, but in terms of the abundance of Lactobacilli and Bifidobacteria, GFD fails to restore the dysbiosis of the gut microbiota in CD patients [8]. Plant‐based diet is also raising concern as a healthy diet with relative high proportion of fruits, vegetables, legumes, and grains, such as vegetarian/vegan diet and the Mediterranean diet. Besides, it is also characterized by applying fish and olive oil as the main sources of protein and fat. Compared to Western diets, plant‐based diets increased the number of fiber‐degrading bacteria and the synthesis of SCFAs and bioactive phenolic acid derivatives, resulting in improved overall health [9]. Similar results have been reported for the DASH diet (which emphasizes the intake of vegetables, fruits, and low‐fat dairy products) and the traditional Mediterranean diet. Certain types of fibers in the DASH diet are thought to be prebiotics, which feed commensal bacteria in the colon and stimulate their growth. Fiber fermentation produces SCFAs, which are used to maintain the intestinal barrier and reduce local inflammation [10]. Mechanisms of dietary patterns and food composition on the gut microbiota remain largely unknown. Considering the enormous impact of diet on the microbiota, a systematic summary of the data on diet‐microbiome‐host interactions is extremely necessary.

## APPLICATION OF GUT MICROBES TO REVOLUTIONIZE HUMAN HEALTH: PROBIOTICS, PREBIOTICS, SYNBIOTICS, AND POSTBIOTICS

Dysbiosis of the gut microbiota can affect the development and function of several major organ systems and can lead to neurological, metabolic, and inflammatory disorders. To address concerns for intestinal health, microecological agents (e.g., probiotics, prebiotics, synbiotics, postbiotics, and parabiotics) are utilized to maintain host microecological balance and improve health, aiding in the prevention and treatment of diseases.

Probiotics are live microorganisms that, if ingested in sufficient amounts, can benefit the host, including the commonly known *Lactobacillus* and *Bifidobacterium* species. Both preclinical and clinical studies have reported that probiotic interventions, which include *Lactobacillus casei* BL23, *Lactobacillus plantarum* A, or a combination of *Bifidobacterium lactis* Bl‐04 and *Lactobacillus acidophilus* NCFM, exhibited a notable antitumor immune effect and were able to restore intestinal microbial balances [11].

Prebiotics are nondigestible food components that act as nutrients for gut microbes, mainly consisting of oligosaccharides, microalgae, and natural plants. Hydrolytic fermentation of prebiotics leads to the production of SCFAs (mainly acetate, butyrate, and propionate), which are beneficial to host health and have been associated with improved insulin sensitivity and reduced inflammation [12]. Glucans and fructans are well documented as prebiotics, and evidence for the prebiotic effects of other substances (e.g., oligomers of mannose, glucose, xylose, and pectin) is growing.

Synbiotics are defined as a combination of prebiotics and probiotics. In numerous randomized controlled trials, synbiotics have been shown to be effective in the prevention and treatment of metabolic diseases, irritable bowel syndrome, surgical infections, chronic kidney disease, and atopic dermatitis [13].

Since many of the health effects of probiotics, prebiotics, or synbiotics depend on the final product of SCFAs and microbial fractions, the concept of postbiotics emerged, which is defined as “the preparation of inanimate microorganisms and/or their components that are beneficial to the host.” Antimicrobials, targeted anti‐inflammatory and immunomodulatory agents, novel signaling molecules affecting intestinal pain, sensation, secretion, and motility, as well as clinical indications to enhance the efficacy of vaccinations or to modulate the immune response, may benefit from potent postbiotic metabolites [14]. Parabiotics are also an emerging concept in the field of functional foods, which are inactivated microbial cells or crude cell extracts of probiotics [15]. Different studies have described the anti‐inflammatory and antioxidant potential of heat‐inactivated *Lactobacillus* strains in in vitro and in vivo models, but there is still a scarcity of available literature on parabiotics [15]. Overall, human resident microorganisms and its specific composition have been associated with many health conditions. How gut microbes can be applied to target human health still needs to be further explored.

## FUTURE EXPLORATION FOR ESTABLISHING PERSONALIZED DIET AND PRECISE NUTRITIONAL INTERVENTION

**Figure 2:**
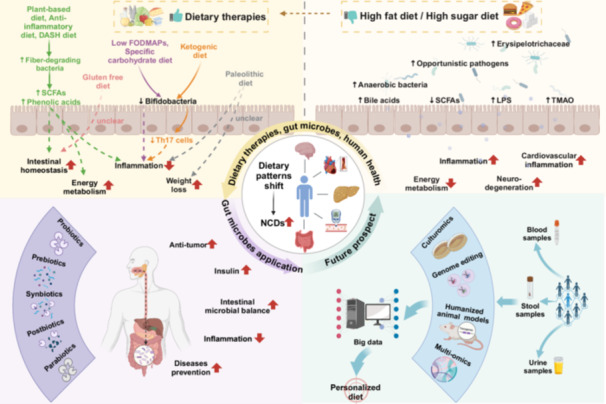
Schematic of future precise personalized diets through integrated linkage of dietary therapies, gut microbes, and human health. Gut microbes, metabolites, and their corresponding pathways toward various dietary patterns are established. On the other hand, the potential application of gut microbes and metabolites for dietary intervention is also presented. Conjoined consensus elicits the prospect of establishing personalized diet and nutritional intervention. DASH, dietary approaches to stop hypertension diet; Low FODMAPs, low‐fermented, oligosaccharide, disaccharide, monosaccharide, and polyol; LPS, lipopolysaccharide; SCFAs, short chain fatty acids; TMAO, trimetlylamine oxide. Figure created with BioRender.com.

Understanding how diet affects gut microbes and thus regulates human health may lead to targeted dietary strategies. Decoding microbial genomes has become essential for revealing their identities. As shown in Figure 2, using advanced technologies such as culturomics, genome editing, humanized animal and organ‐on‐chip models, and multi‐omics, our understanding of the function of the gut microbiota can be further enhanced [16]. Revealing the interaction between diet and gut microbiome through these techniques under different dietary interventions may provide assistance in the development of personalized diets. Personalized nutritional services for targeted individuals, including those with obesity, diabetes, gastrointestinal diseases, neurodegenerative diseases, and cardiovascular diseases, hold promising commercial potential.

With changing lifestyles and dietary patterns, research has shifted from nutritional deficiencies to NCDs [17]. WHO dietary guidelines recommended nutrient‐dense foods and beverages with sufficient vitamins, minerals, and other health‐promoting ingredients, while minimizing added sugars, saturated fats, and sodium to reduce the risk of chronic diseases [18]. However, these guidelines do not account for interpersonal variation in nutritional requirements. Personalized diets can offer more specific guidance on healthy eating and nutritional products based on an individual's genetic, phenotypic, medical, nutritional, and other relevant information. Personalized dietary advice may ultimately assist and motivate people to follow a healthier diet and lifestyle. Furthermore, big data has been proposed to make more accurate and effective dietary recommendations to individuals. Algorithms can be based on a variety of inputs, including an individual's tastes and preferences, clinical characteristics, and other factors (e.g., gut microbiota and genetics). This process needs to be supported by behavioral and biological evidence [19]. For example, in a precision health longitudinal big data study, the cohort performed a comprehensive personalized omics profiling of collected samples using clinical measures and emerging technologies, including multi‐omics and wearable monitoring. The study identified multiple molecular pathways associated with metabolic, cardiovascular, and tumor pathophysiology and developed a predictive model for insulin resistance. Research participation ultimately led to the implementation of dietary and exercise changes for most participants [20]. However, the supporting data of gut microbiomics and genetic and metabolomic information for personalized dietary advice remain lacking. Considering the complexity of interpersonal variations in diet‐microbe‐host interactions, incorporating metabolomic and gut microbiomics analyses of different groups may provide valuable insights for developing successful personalized diets. Overview, we believe that advancement of human microbiome and bio‐medical research will revolutionize the precise personalized dietary therapies. With the development of artificial intelligence (AI), integrating database will further prospect for personalized dietary therapies with high precision.

## AUTHOR CONTRIBUTIONS

**Jiali Chen and Wah Yang**: Writing—original draft. **Jiali Chen**: Conceptualization. **Jiaqiang Luo**, **Sjaak Pouwels**, **Beijinni Li**, **Bian Wu**, **Tamer N. Abdelbaki**, **and Jayashree Arcot**: Writing—review and editing. **Jiali Chen and Wah Yang**: Supervision. All authors have read the final manuscript and approved it for publication.

## CONFLICT OF INTEREST STATEMENT

The authors declare no conflict of interest.

## ETHICS STATEMENT

No animals or humans were involved in this study.

## Data Availability

Data sharing is not applicable to this article as no datasets were generated or analyzed during the current study. This manuscript does not generate any code or data. Supplementary materials (graphical abstract, slides, videos, Chinese translated version and update materials) may be found in the online DOI or iMeta Science http://www.imeta.science/.
